# *Streptococcus Constellatus* Spondylodiscitis in a Teenager: A Case Report

**DOI:** 10.5704/MOJ.1711.004

**Published:** 2017-11

**Authors:** SW Lim, HY Lim, T Kannaiah, Z Zuki

**Affiliations:** Department of Orthopaedics, Hospital Sungai Buloh, Sungai Buloh, Malaysia

**Keywords:** Streptococcus constellatus, S. constellatus spondylodiscitis, CT guided biopsy, low back pain

## Abstract

*Streptococcus constellatus* is an extremely rare cause of pyogenic spondylodiscitis. Literature search yielded only one case report in an elderly 72 years old man with spontaneous T10-T11 *S. constellatus* spondylodiscitis. It is virtually unheard of in young teenage. We report the case of a 14 years old male teenager who presented with worsening low back pain for one year with no neurological deficit. Imaging studies were consistent with features of L4-L5 spondylodiscitis. CT guided biopsy grew a pure culture of *streptococcus constellatus* sensitive to penicillin and erythromycin. He showed full recovery with six weeks of intravenous antibiotics. Due to the insidious onset, this case highlight the importance of high clinical suspicion and early diagnosis, with image guided biopsy followed by treatment with appropriate intravenous antibiotics to enable full recovery without further neurological deterioration.

## Introduction

Infective spondylodiscitis accounts for 2-7% of all cases of musculoskeletal infections^1^. Spine infection is caused by three major agents: bacteria, causing pyogenic infection; tuberculosis or fungi, responsible for granulomatosis infection; or parasites, which are the least common etiology. The majority of spinal infections are bacterial monomicrobial caused by Staphylococcus aureus with an incidence between 30 and 80%^2^. Gram-negative bacteria, such as Escherichia coli, are responsible, in some series, for up to 25% of spinal infection^2^. *Streptococcus constellatus* infection is an extremely rare cause of pyogenic spondylodiscitis; literature search yielded only one case report in an elderly 72 years old man^3^ and is virtually unheard of in young healthy teenagers.

## Case Report

We report the case of a 14 years old male teenager who presented with gradually worsening intermittent localized dull aching low back pain which was aggravated by prolonged standing, sitting or walking, for the past one year. It was associated with significant weight loss of more than 10kg during that period and loss of appetite. There was history of night sweats, evening rise of temperature as well as multiple episodes of sinuses over the lower back and gluteal region with pus discharge which had resolved for a few months prior to current presentation. There was no history of persistent cough, tuberculosis contact, high risk behaviour or trauma to the lower back. There were no altered bowel or bladder habits. There was no history of dental abscess or procedures done previously. He had no known past medical illness and no previous surgery, blood transfusion or trauma. He lived with his parents along with four siblings and there was no family history of tuberculosis or malignancy. Tuberculosis workout had been done in a district hospital and reported negative. He was referred to a tertially hospital with spine service when there was worsening of low back pain despite management with analgesia and physiotherapy.

**Fig. 1:**
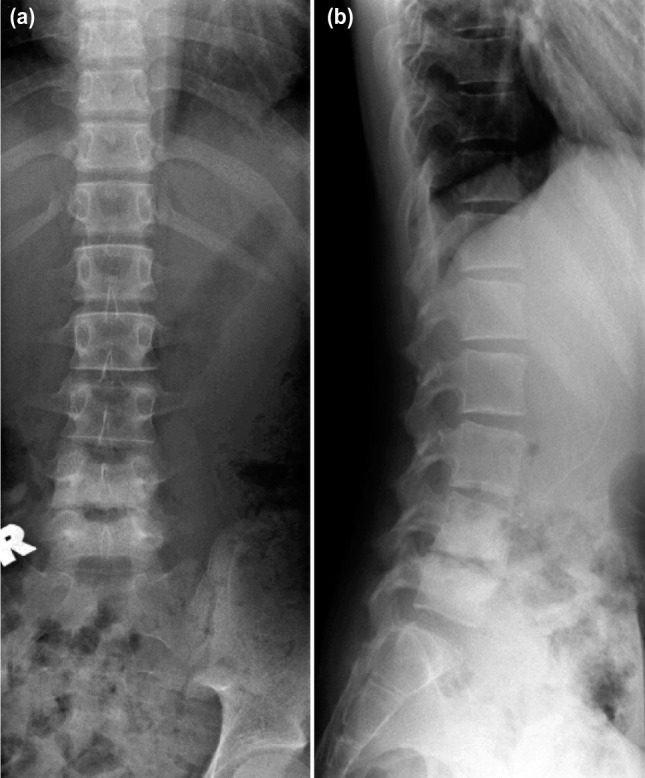
(a) Radiograph of lumbosacral anteroposterior view showing reduced joint space with end plate erosion over L4-L5 region and (b) Radiograph of lumbosacral lateral view showing reduced joint space, end plate erosion over L4-L5 vertebra and also loss of lumbar lordosis.

**Fig. 2:**
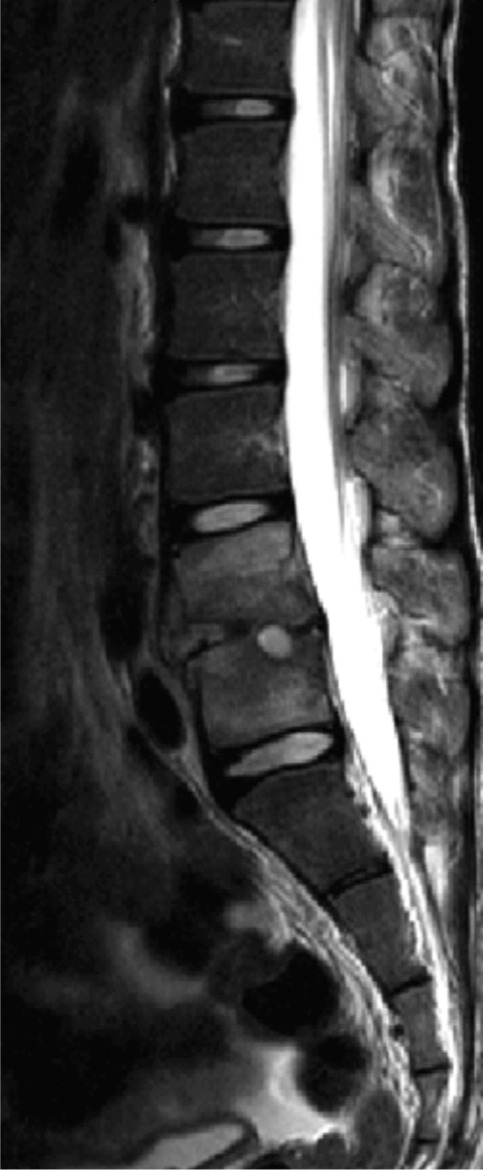
MRI mid sagittal T2 weighted image of the lumboscral spine showing L4-L5 intervertebral disc destruction.

On examination, there was minimal tenderness over the lower lumbar region on palpation. There were no spinal deformity or gibbus. Range of motion of lumbar spine was restricted due to pain. Neurological examination revealed no motor or sensory deficit. Reflexes were normal over both upper and lower limbs. Examination of cardiovascular, respiratory and gastrointestinal systems revealed no abnormalities. Laboratory investigation revealed raised Erythrocyte Sedimentation Rate (ESR) of 69 mm/hr (the normal range is 0-22 mm/hr for men and 0-29 mm/hr for women), C-Reactive Protein (CRP) of 0.7 mg/dL (Normal values are below 3.0 mg/dL) and White Cell Count of 9.43× 10^9^/L (Normal values range from 4.5 to 11.0 x 10^9/L). Mantoux test and sputum for Acid Fast Bacilli (AFB) were negative on three occasions. Chest radiograph was normal. Radiographs of the lumbosacral spine revealed collapse and reduction in joint space with erosion of end plates over L4-L5 region and loss of lumbar lordosis (Fig. 1). An MRI of the lumbosacral spine showed loss of L4-L5 disc space with minimal collection of extraneous material with adjacent paravertebral extension (Fig. 2). A CT guided biopsy of L4/L5 disc level yielded only obtained a small soft tissue sample which grew a pure culture of *Streptococcus constellatus* which was sensitive to penicillin and erythromycin biopsy was not repeated.

He was treated with intravenous C-penicillin 4 mega unit 4-hourly for total duration of six weeks after consultation with infectious disease team. He recovered well with gradual resolution of back pain and his inflammatory markers returned to normal just two weeks after initiation of treatment. The patient achieved full recovery after completion of six weeks of intravenous antibiotics.

## Discussion

*Streptococcus contellatus* along with *S. intermedius* and *S. anginosus* are collectively referred to as the *S. milleri* group. It is a gram-positive group of C-beta haemolytic microaerophyllic streptococci that form part of the normal flora in the oral cavity, urogenital region and intestinal tract but known to cause upper body abscesses and respiratory infections, especially in immunocompromised individuals ^4^. It has also been found to be involved with pulmonary exacerbations in cystic fibrosis patients^5^.

Pyogenic spondylodiscitis caused by *S. constellatus* is virtually unheard of. Literature search yielded only a single case report by Gangone *et al* in 2009 in which a 72 years old man with a history of worsening back pain was diagnosed with T10-T11 *S. constellatus* spondylodiscitis from CT guided biopsy and he made a full recovery with treatment of intravenous antibiotics and bed rest. To our knowledge, ours is the only case of *S. constellatus* spondylodiscitis that occurred spontaneously in the lower lumbar spine in an otherwise healthy young male teenager. As it is microaerophyllic, the organism can survive well in an oxygen-depleted environment causing subacute spondylodiscitis. Due to the insidious onset, this case highlights the importance of high clinical suspicion and early diagnosis with image guided biopsy followed by treatment with appropriate intravenous antibiotics to enable full recovery without further neurological deterioration.
